# On the Bipolar DC Flow Field-Effect-Transistor for Multifunctional Sample Handing in Microfluidics: A Theoretical Analysis under the Debye–Huckel Limit

**DOI:** 10.3390/mi9020082

**Published:** 2018-02-16

**Authors:** Weiyu Liu, Qisheng Wu, Yukun Ren, Peng Cui, Bobin Yao, Yanbo Li, Meng Hui, Tianyi Jiang, Lin Bai

**Affiliations:** 1 School of Electronics and Control Engineering, Chang’an University, Middle-Section of Nan’er Huan Road, Xi’an 710064, Shanxi, China; liuweiyu@chd.edu.cn (W.L.); b.b.yao@chd.edu.cn (B.Y.); ybl@chd.edu.cn (Y.L.); ximeng@chd.edu.cn (M.H.); linbai@chd.edu.cn (L.B.); 2 State Key Laboratory of Robotics and System, Harbin Institute of Technology, West Da-zhi Street 92, Harbin 150001, Heilongjiang, China; shadowwalker2014@163.com (P.C.); jhy_hit@hit.edu.cn (T.J.)

**Keywords:** bipolar DC field-effect flow control, flow field-effect-transistor, counterionic Debye screening, simultaneous electroconvective pumping and mixing in microfluidics, linear electroosmosis, induced-charge electroosmosis

## Abstract

We present herein a novel method of bipolar field-effect control on DC electroosmosis (DCEO) from a physical point of view, in the context of an intelligent and robust operation tool for stratified laminar streams in microscale systems. In this unique design of the DC flow field-effect-transistor (DC-FFET), a pair of face-to-face external gate terminals are imposed with opposite gate-voltage polarities. Diffuse-charge dynamics induces heteropolar Debye screening charge within the diffuse double layer adjacent to the face-to-face oppositely-polarized gates, respectively. A background electric field is applied across the source-drain terminal and forces the face-to-face counterionic charge of reversed polarities into induced-charge electroosmotic (ICEO) vortex flow in the lateral direction. The chaotic turbulence of the transverse ICEO whirlpool interacts actively with the conventional plug flow of DCEO, giving rise to twisted streamlines for simultaneous DCEO pumping and ICEO mixing of fluid samples along the channel length direction. A mathematical model in thin-layer approximation and the low-voltage limit is subsequently established to test the feasibility of the bipolar DC-FFET configuration in electrokinetic manipulation of fluids at the micrometer dimension. According to our simulation analysis, an integrated device design with two sets of side-by-side, but upside-down gate electrode pair exhibits outstanding performance in electroconvective pumping and mixing even without any externally-applied pressure difference. Moreover, a paradigm of a microdevice for fully electrokinetics-driven analyte treatment is established with an array of reversed bipolar gate-terminal pairs arranged on top of the dielectric membrane along the channel length direction, from which we can obtain almost a perfect liquid mixture by using a smaller magnitude of gate voltages for causing less detrimental effects at a small Dukhin number. Sustained by theoretical analysis, our physical demonstration on bipolar field-effect flow control for the microfluidic device of dual functionalities in simultaneous electroconvective pumping and mixing holds great potential in the development of fully-automated liquid-phase actuators in modern microfluidic systems.

## 1. Introduction

**Figure 1:**
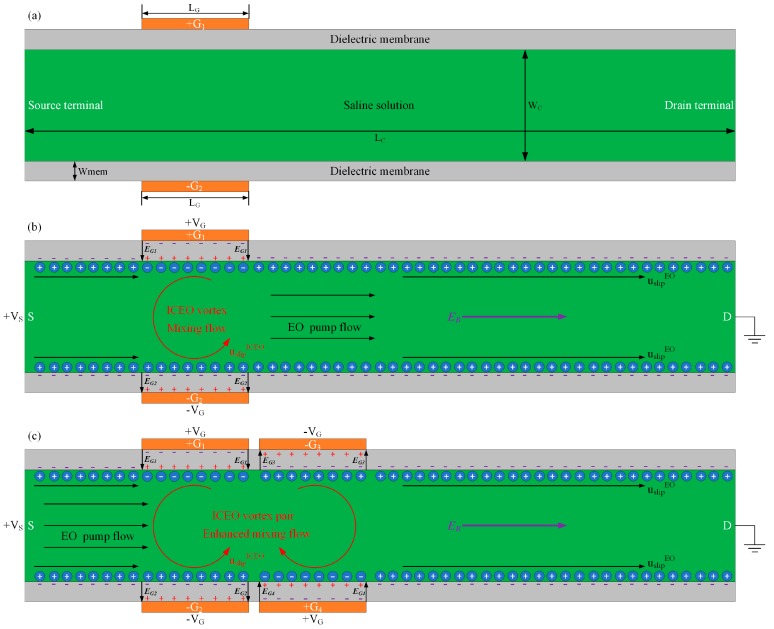
2D schematic diagrams of the microfluidic device for bipolar DC field-effect electroosmosis control. (**a**) Geometric configuration of a bipolar DC flow field-effect-transistor (DC electroosmosis (DCEO)), in which one or several pairs of face-to-face gate (G) terminals of counter voltage polarities are embedded in a straight microchannel on the external surfaces of both sidewalls. (**b**,**c**) A vivid illustration of the physicochemical mechanism responsible for causing simultaneous sample pumping and mixing with different discrete arrangements of the external gate electrode array; (b) in the structural configuration of one pair of face-to-face oppositely-polarized gate-electrode, an individual induced-charge electroosmotic (ICEO) vortex is induced across the channel width direction next to the two G terminals; and (c) in the microfluidic device of two neighboring sets of upside-down G terminal pairs, a pair of ICEO whirlpools in reversed rotating directions is induced in the vicinity of the four external gate electrodes. Interestingly, in (c) the pair of symmetric counter-rotating ICEO micro-vortices is induced by nonlinear electroosmotic slip on the surface of polarizable membranes, within which strong electric field leakage occurs due to the action of external G terminals, and the electrokinetics-driven turbulence adds effectively to the horizontal DCEO flow component in the forward direction to achieve simultaneous pumping and mixing of electroneutral chemical analytes in microfluidics.

The advent of micrometer-scale fluidic technology prompts the fundamental issue of how to deliver and mingle fluidic samples at the micrometer dimension, where Poiseuille flows and hydrodynamic instabilities from inertia effects are severely suppressed by liquid viscosity [1,2,3,4,5,6,7,8]. The most prevalent non-mechanical pumping approach is based on conventional DC electroosmotic flow [9]. For microfluidic channels full of conducting saline solutions, a surface charge density, usually of negative polarity, can be adsorbed on channel sidewalls through some kind of physicochemical process, resulting in a thin free surface charge layer on the dielectric membranes, as shown in the non-covered area of gate (G) terminals in Figure 1b. Due to normal field lines emitted from the fixed surface charge layer on channel sidewalls, a dynamic balance between electrostatic attraction and surface ion diffusion from diffuse charge dynamics induces a distribution of counterionic charge in a thin electrical double layer (EDL) at the solution/membrane interface to compensate for the fixed surface charge to maintain global electroneutrality [10,11,12,13,14,15,16,17]. Since it is originated by a fixed surface charge layer, this kind of diffuse screening cloud is named native EDL, which occurs spontaneously independent of any other applied voltages. Under this situation, a tangential field component imposed across the source (S) and drain (D) terminals acts on the volumetric free charge within the thin EDL, and the resulting Coulomb force can effectively actuate the mobile counterions into unidirectional DC electroosmosis (DCEO) streaming flow along the channel length direction for active pumping of fluid samples (Figure 1b,c).

The Debye screening length of counterions has a characteristic distance scale *λ*_D_ = $\sqrt{D\varepsilon_{f}/\sigma_{f}}$ away from the charged channel sidewalls [18,19,20,21,22]. Here, D = 2 × 10^−9^ m^2^/s denotes the diffusivity of medium charge carriers, with *ε_f_* and *σ_f_* being the dielectric permittivity and electrical conductivity of the working fluid, respectively. In this sense, the thickness of EDL decreases sharply with an increase in liquid conductivity and becomes even less than several nanometers for a sufficiently strong ionic strength. The severe suppression of double-layer extension in concentrated solutions makes the fixed zeta potential *ξ*_fixed_ across the diffuse part of native EDL greatly reduced, resulting in much weakened electroosmotic slip velocity at the outer edge of the Debye layer [23,24,25].

To overcome this shortcoming of traditional electroosmosis at higher ionic conductivities, Schasfoort et al. [26] innovatively developed a series connection model of three-capacitors at the end of the last century, including the dielectric membrane, compact layer and Debye screening cloud, to introduce a genuinely new physical concept of driving stronger electroosmotic flow in microfluidic networks, and coined the term ‘microscale field-effect flow control’ to delineate this general class of device configurations of flow field-effect transistors for regulating electroosmotic flows, in analogy to the typical structure of semiconductor transistors for modulating output electric currents. In this three-electrode-based microfluidic device, one individual gate (G) terminal is deposited by microelectronic processing technology on the level of the outer surface of polarizable dielectric membranes in direct contact with the micro-scale duct. A DC gate voltage is routinely applied to the external G terminal to produce extra induced Debye screening charge across the diffuse part of an induced double-layer capacitance at the membrane/solution interface, with the induced zeta potential effectively superimposed on the native zeta potential from the fixed free surface charge. A background static DC electric field applied across the channel axial direction compels both the native and induced counterionic cloud into electroosmotic streaming, resulting in acceleration of the electrokinetics-driven pump flow rate [27,28,29], compared to the conventional electroosmosis without the induced-charge electrokinetic phenomenon (ICEK) [30,31,32,33,34,35] from externally-arranged gate terminals. Before long, the leakage current density flowing across the three capacitors in series connection was incorporated into a modified model of the flow field-effect-transistor (flow-FET) by the group of Dutta [36], to enable a better comprehension of field-effect electroosmosis control in microfluidic networks. Flow-FET has received considerable attention from the microfluidic community over the past two decades [37,38,39,40,41,42,43,44,45]. Most works on this interesting subject have focused on how to improve the pump flow rate from DCEO [46,47], while other kinds of electrokinetic sample manipulations with flow-FET are less of concern for micro-/nano-fluid dynamicists. 

To enable more comprehensive functionalities of flow-FET, we proposed herein a modified version of the previous flow-FET with merely a unipolar gate terminal and coined the term ‘bipolar DC field-effect flow control’ or ‘bipolar DC flow field-effect transistor (B-DCFFET)’, as shown in Figure 1. In stark contrast to the conventional electroosmosis with merely parallel streamlines along the channel length direction, turbulent induced-charge electroosmotic (ICEO) [48,49,50,51,52,53,54] micro-vortices appear in the lateral direction, as well. A pair of face-to-face gate (G) terminals of reversed gate-voltage polarities are embedded in a straight microchannel on both sidewalls, which contributes to inducing the in-phase Debye screening charge of opposite charge polarities inside the thin double layer next to the face-to-face oppositely-polarized G terminals, respectively.

The field-induced face-to-face ionic charge of opposite polarities within the diffuse screening cloud is then forced by a DC voltage difference applied across the source-drain terminal into a large ICEO vortex flow in the transverse direction. Accordingly, the lateral ICEO whirlpool intersects perpendicularly with unidirectional plug flow of DCEO, giving rise to distorted electroosmotic streamlines, which are of dual functionalities in terms of simultaneous DCEO pumping and ICEO mixing of chemical analytes toward the outlet port.

To enable a better comprehension of bipolar field-effect flow control introduced in this work, we developed a mathematical model under the Debye–Huckel limit to correctly capture the resulting flow field from DCEO pumping throughout the channel length direction and the lateral ICEO vortex flow field adjacent to the external G terminals of opposite polarities. For microscale gate electrodes and dilute electrolytes, thin-layer approximation can be safely invoked, to analyze the electrohydrodynamic (EHD) flow field due to a combined effect of the gating-field for inducing additional counterions and the source-drain-field for acting on these double-layer charges. Debye screening in the saline solution is taken into consideration to account for Coulomb force within both the native and induced double layer that propels DCEO plug flow and rotatory ICEO vortexes in the vicinity of ideally-polarizable surfaces, respectively.

A linear asymptotic analysis is subsequently conducted to evaluate the feasibility of the B-DCFFET configuration in electrokinetic manipulation of fluids at the micrometer dimension. According to our simulation analysis, in an integrated device structure with two sets of side-by-side, but upside-down gate-electrode pairs, a pair of counter-rotating ICEO micro-eddies is actuated under the covered area of four G terminals (Figure 1c). A subtle superposition of parallel DCEO streamlines along the channel length direction and ICEO vortex pair in the transversal direction would be of great benefit to achieve simultaneous pumping and mixing of fluids at the microscale. Moreover, a microdevice paradigm is established with multiple sets of reversed bipolar gate-terminal pairs arranged along the top surface of the dielectric membrane on both sides of the microchannel and exhibits very good performance in simultaneous pumping and mixing of microflows for fully electrokinetics-driven analyte treatment. This highly-integrated arrangement of gate terminal arrays of alternating voltage polarities makes it possible for us to fabricate complex microfluidic networks, from which we can obtain almost perfect liquid mixture at the downstream channel exit even in the absence of artificially-imposed pressure gradients, which supplies invaluable physical perspectives to the construction of smart on-chip liquid-phase platforms for fully-automated sample handling in contemporary microfluidic systems.

## 2. Methods

### 2.1. Device Design of B-DCFFET

In this work, we conduct a simulation analysis on the DCEO flow field in the device utilizing the phenomenon of the Bipolar DC Flow Field-Effect-Transistor (B-DCFFET), which has been defined in the Introduction section. In such a device design, one or several progressively-shifted sets of face-to-face bipolar gate electrode pairs of opposite voltage polarities are embedded on both sides of the channel sidewalls, as shown in Figure 1. The basic device structure for driving B-DCFFET next to polarized coatings in a straight microchannel is exhibited in Figure 1a, where the central channel is enclosed by dielectric films on both sides.

Static DC gate voltages ‘V_G_, −V_G_, V_G_, −V_G_…’ of consecutively alternating polarities are applied to the G terminal array on the left side in sequence, while the reversed voltage polarities ‘−V_G_, V_G_, −V_G_, V_G_…’ are imposed on its counterpart on the right side, as shown in Figure 1c. In this way, an applied DC voltage difference *Vs* across the S-D terminal forces the field-induced bipolar counterionic charge adjacent to these G terminals into a series of ICEO bulk vortexes in alternative rotating directions along the channel length direction. Under this circumstance, the lateral turbulent stirring flow of ICEO and the longitudinal DCEO transport flow are effectively added to one another to form helical electroosmotic streamlines in the forward direction, resulting in simultaneous electroconvective pumping and mixing in the microfluidic device of B-DCFFET. 

The straight microchannel of L_c_ = 2500 μm in length and W_c_ = 200 μm in width and the dielectric films of L_PDMS_ = 2500 μm in length and W_PDMS_ = 10 μm in width are simultaneously deposited onto the top surface of an insulating glass substrate, all of which have an identical height to guarantee watertight bonding. The microfluidic channel is assumed to be full of aqueous electrolyte solution of conductivity *σ_f_* = 0.001 s/m and permittivity *ε_f_* = 80 *ε*_0_. As we are looking for the optimal parametric space to engender the most effective electrokinetics-driven helical streamlines toward the downstream by conducting direct numerical simulation with the control variate method, the feasibility of B-DCFFET in simultaneous electroconvective pumping and mixing of fluid samples is verified within a broad parametric space. In this microfluidic pump and mixer, two upstream channel inlets and one downstream outlet lead to three macroscale reservoirs, respectively. The entrance may be of a ‘Y’ shape. According to our simulation results, B-DCFFET is able to electro-convectively pump and mix the chemical analytes to be processed at the same time, and there is no need to apply an external pressure difference to produce forward laminar streaming, which gives rise to a smart microscale liquid-phase platform for automatic electrokinetic sample treatment.

### 2.2. Mathematical Model

Our mathematical depiction of simultaneous pumping and blending of fluidic samples from B-DCFFET is on the basis of the linear circuit theory of electrochemical polarization in the small-voltage limit. From a macroscopic scale, we can deal with the boundary-value problem by partitioning the whole device into three correlated compartments, including the solution domain, the thin induced double-layer, as well as the insulating membranes in immediate contact with microchannel sidewalls on both sides. 

Under the Debye–Huckel limit, the electrostatic potential field in the bulk fluid and dielectric membrane are both governed by the Laplace equation with zero volumetric free charge density [55,56,57,58,59]:

(1) $$\nabla^{2}\phi_{f}=0$$

(2) $$\nabla^{2}\phi_{\mathrm{ins}}=0$$

At the membrane/solution interface, we must provide conjugating conditions to describe induced double-layer dispersion as an interfacial capacitance between the fluid and dielectric membrane [60]:

(3) $$n\cdot \nabla \phi_{f}=0$$

(4) $$\varepsilon_{\mathrm{ins}}n\cdot \nabla \phi_{\mathrm{ins}}=\frac{C_{D}}{1+\delta }\left(\phi_{f}-\phi_{\mathrm{ins}}\right)$$

where *n* denotes the unit normal vector at the membrane surface, *δ* = *C*_D_/*C*_s_ the ratio of diffuse double-layer capacity *C*_D_ = *ε*_f_/*λ*_D_ to Stern layer capacitance *C*_s_ = 0.8 F/m^2^, both of which are in series connection, and *λ*_D_ = $\sqrt{D\varepsilon_{f}/\sigma_{f}}$ the Debye screening distance, with D = 2 × 10^−9^ m^2^/s denoting the diffusivity coefficient of medium charge carriers. Equations (3) and (4) indicates implicitly that the diffusion flux and electromigration flux counterbalance each other within the thin boundary layer, in that ionic species are not able to go through the liquid/membrane interface from physical constraints. As a consequence, the normal conduction current fades away inside the Debye screening cloud. Accordingly, the total Ohmic current from the conducting electrolyte vanishes at the outer edge of the Debye screening cloud due to complete charging of the induced double layer in DC fields (Equation (3)). Additionally, the electrical displacement vector has to be continuous at the interface between the double layer and dielectric membrane (Equation (4)), so as to guarantee charge conservation in static fields.

In the current analysis, we employ the superposition principle in electrochemistry to separately consider the effect of the native double-layer charge and its induced counterpart. The two distinct kinds of zeta potentials are determined as follows:

(5) $$\zeta_{\mathrm{fixed}}=\frac{\sigma_{\mathrm{free}}}{C_{D}}$$

(6) $$\zeta_{i}=\frac{\phi_{\mathrm{ins}}-\phi_{f}}{1+\delta }$$

DC voltage magnitudes are set to given values at the electrode terminals for field-effect electroosmosis control at the micrometer dimension:

(7) $$\phi_{f}=V_{s}\;(\text{S terminal at the channel entrance})$$

(8) $$\phi_{f}=0\;(\text{D terminal at the channel exit})$$

(9) $$\phi_{G}=V_{G}\;\mathrm{or}\;\phi_{G}=-V_{G}\;(\text{G terminal})$$

The normal current flux disappears at the outer surface of the membrane due to the negligible polarizability of the air medium in the environment:

(10) $$n\cdot \nabla \phi_{f}=0$$

The tangential electric field component $E_{t}=-\nabla \phi_{f}$ exerts ponderomotive Coulomb forces within the native and induced double layers at the same time, resulting in slip velocity of linear and nonlinear electroosmosis along the channel sidewall, respectively:

(11) $$u_{\mathrm{slip}}^{\mathrm{EO}}=-\frac{\varepsilon_{f}\varsigma_{\mathrm{fixed}}}{\eta }E=-\frac{\varepsilon_{f}}{\eta }\frac{\sigma_{\mathrm{free}}}{C_{D}}E$$

(12) $$u_{\mathrm{slip}}^{\mathrm{ICEO}}=-\frac{\varepsilon }{\eta }\zeta_{\mathrm{induced}}E=-\frac{\varepsilon }{\eta }\frac{\left(\phi_{\mathrm{ins}}-\phi_{f}\right)}{1+\delta }E$$

Therefore, the total electroosmotic slip velocity at the membrane/electrolyte interface is given by the sum of Equations (11) and (12):

(13) $$u_{\mathrm{slip}}^{}=-\frac{\varepsilon_{f}}{\eta }\frac{\sigma_{\mathrm{free}}}{C_{D}}E-\frac{\varepsilon }{\eta }\frac{\left(\phi_{b}-\phi_{f}\right)}{1+\delta }E$$

We then calculate the bulk fluid motion driven by electroosmosis through inserting Equation (13) into the full Stokes equation as a slip boundary condition on channel sidewalls:

(14) $$-\nabla p+\eta \nabla^{2}u=0$$

(15) ∇⋅u=0

where *p* stands for the hydrodynamic pressure field and *u* the ICEO flow field.

The convection-diffusion equation is employed in the current work to numerically obtain the molar concentration distribution of sample nanoparticles, due to the combined action of diffusive mass transfer and electroconvective mass transport from both longitudinal DCEO pump flow and lateral ICEO vortex turbulence:

(16) $$\nabla \cdot \left(uc-D_{\mathrm{solute}}\nabla c\right)=0$$

where *c* represents the local concentration and *D*_solute_ = 10^−11^ m^2^/s the Brownian diffusivity of the nano-colloids suspended in buffer solution, which is determined by the Einstein relation for nanospheres with a radius of r = 20 nm.

### 2.3. Numerical Simulation

A commercial FEM-based software package, Comsol Multiphysics, is utilized in this work to study B-DCFFET with face-to-face oppositely-polarized G terminals, as well as its application in simultaneous electroconvective pumping and mixing of fluid samples in microfluidics. The simulation procedure of electrokinetic fluid motion and resulting mass transfer in the device configuration of B-DCFFET are as follows:

(a) Firstly, the two Laplace equations, including Equations (1) and (2), are calculated to obtain the electrostatic potential in the buffer medium and polydimethylsiloxane (PDMS) channel sidewalls, respectively. DC voltage signals $\phi_{f}=V_{s}$ and $\phi_{f}=0$ are designated on the S terminal at the entrance and the D terminal at the outlet, respectively. Static gate potentials $\phi_{G}=V_{G}$ or $\phi_{G}=-V_{G}$ are applied to the bipolar gate electrode array embedded on both sides of the microdevice. The appearance of induced counterionic charge is reflected by the joint conditions (Equations (3) and (4)) at the solution/membrane interface. Besides, the zero normal current component $n\cdot \nabla \tilde{\phi }=0$ is given at the membrane/air interface to close the electrostatic boundary-value problem.
(b) Secondly, the full Stokes Equations (14) and (15) are computed to obtain the velocity field of electroconvective streaming in B-DCFFET, with the superimposed slip velocity from linear and nonlinear electroosmosis (Equation (13)) preset at the saline-solution/membrane interface on both sides. The channel inlet and outlet are both set to open boundaries for describing the phenomenon of simultaneous electroconvective pumping and mixing, which are fully originated by electroosmotic flow.
(c) Thirdly, convection-diffusion Equation (16) is calculated to resolve the density distribution of chemical analytes within the saline solution under the impact of both diffusive and electroconvective mass transfer. Normal flux vanishes at the phase interface. Current work employs fluorescein of 40 nm in diameter of diffusivity D = 10^−11^ m^2^·s^−1^ as the fluid samples. Analyte concentration c = 1 mol·m^−3^ and c = 0 mol·m^−3^ is fixed at the left and right side of the channel entrance, respectively, and diffusion flux disappears at the channel exit.

Stationary solvers are employed for solving this set of governing equations subjected to physically-justifiable boundary and conjugate conditions. The static electric field, electroosmotic flow and sample delivery are solved sequentially in a segregated way. A minimum mesh size of 2.5 μm is set on the membrane surface, with a maximum growth rate of 1.03 for the extension of grids from both sides to the central fluid bulk, which makes a great contribution to grid independence. An approximate analytical solution for induced zeta potential is derived, which helps validate the effectiveness of our simulation model (please refer to the Supplementary Material).

### 2.4. Scaling Analysis

By conducting an impedance analysis in static fields, the magnitude of induced zeta potential scales as:

(17) $$\zeta_{\mathrm{induced}}\propto V_{G}\cdot \frac{\varepsilon_{\mathrm{ins}}\lambda_{D}}{\varepsilon_{f}W_{\mathrm{ins}}}$$

In a similar way, the native zeta potential from fixed surface charge density chemically on channel sidewalls scales as:

(18) $$\zeta_{\mathrm{fixed}}\propto \frac{\sigma_{\mathrm{free}}\lambda_{D}}{\varepsilon_{f}}$$

Therefore, according to Equations (11) and (12), the scaling characteristics of the DCEO pump flow and ICEO mixing flow components are respectively given by:

(19) $$u_{\mathrm{DCEO}}^{\mathrm{pump}}\propto \frac{\sigma_{\mathrm{free}}\lambda_{D}V_{S}}{\eta L_{C}}$$

(20) $$u_{\mathrm{ICEO}}^{\mathrm{mixing}}\propto \frac{V_{G}V_{S}\varepsilon_{\mathrm{ins}}\lambda_{D}}{\eta W_{\mathrm{ins}}L_{C}}$$

Equation (20) implies that both V_G_ and V_S_ make a contribution toward inducing the interfacial counterionic charges and in turn drive their own induced Debye screening charge into ICEO streaming, which is clearly different from traditional DCEO flow, which is linearly proportional to the applied electric field (Equation (19)).

Taking into account the corresponding role of linear and nonlinear electroosmosis in electrokinetic sample treatment, the device mixing performance can be qualitatively decided by the ratio of these two electroconvective flow components:

(21) $$\beta =\frac{u_{\mathrm{ICEO}}^{\mathrm{mixing}}}{u_{\mathrm{DCEO}}^{\mathrm{pumping}}}=\frac{\zeta_{\mathrm{induced}}}{\zeta_{\mathrm{fixed}}}=\frac{\varepsilon_{\mathrm{ins}}V_{G}}{\sigma_{\mathrm{free}}W_{\mathrm{ins}}}=\alpha$$

Accordingly, to get a higher mixing index with B-DCFFET, we ought to intensify the vortex flow field of ICEO turbulence $u_{\mathrm{ICEO}}^{\mathrm{mixing}}$, or in other words, enhance the induced zeta potential $\zeta_{\mathrm{induced}}$ by increasing the gate voltage magnitude $V_{G}$ and/or raising the polarizability of the dielectric membrane (a higher value of $\varepsilon_{\mathrm{ins}}/W_{\mathrm{ins}}$).

### 2.5. Mixing Index

The classical definition of the mixing index γ is used herein to appraise the device mixing performance at the channel exit, with electroconvective pumping and mixing by B-DCFFET at the same time:

(22) $$\gamma =\left(1-\frac{\iint_{S}\left|c-0.5[\mathrm{mol}/m^{3}]\right|dA}{\iint_{S}0.5[\mathrm{mol}/m^{3}]dA}\right)\times 100\%$$

where c denotes the analyte molar concentration in the outlet plane.

## 3. Results and Discussion

**Figure 2:**
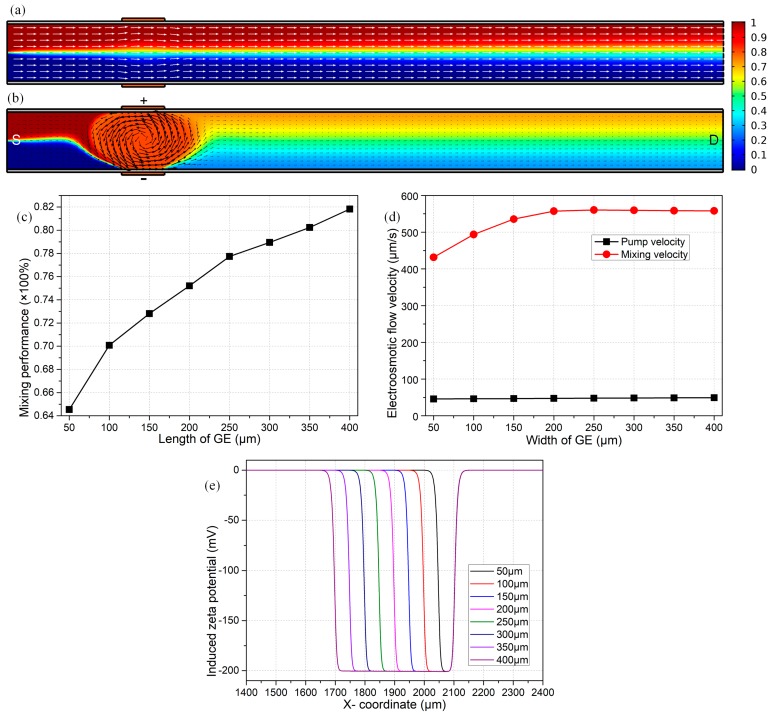
Effect of the length of gate terminal L_G_ on the device performance in terms of dual functionalities. (**a**,**b**) An arrow and surface plot of the electroosmotic flow field and analyte concentration distribution in the straight microchannel. (a) Passive mixing of incoming samples carried by a forward electroosmotic pump flow rate of 47 μm/s under Vs = 30 V in the absence of gate voltage supplies, where merely the molecular diffusion effect in the vertical concentration gradient across the phase interface between the two co-flowing laminar streams occurs and is responsible for the passive mixing process, resulting in a poor mixing index of 27.8%. (b) Active mixing is actuated with all the DC voltage terminals turned on, in which the pump flow along the channel axial direction is caused by DC electroosmosis, and the lateral turbulent mixing flow is originated by ICEO from Bipolar DC Flow Field-Effect Transistor (B-DCFFET) with a GE length of L_G_ = 150 μm, for given values of V_S_ = 30 V, V_G1_ = −V_G2_ = 1500 V, giving rise to an enhanced mixing performance of γ = 72.81%. (**c**) L_G_-dependent device mixing performance. (**d**) Electroosmotic pump and mixing flow velocity as a function of the width of the G terminal. (**e**) Distribution of the induced zeta potential at the solution/membrane interface adjacent to the gate electrode with different L_G_; the negative sign indicates positive counterionic charges in the vicinity of the negatively-polarized G terminal.

To start with, we concentrate on the electroosmotic flow pattern on the membrane surface in the device configuration exploiting B-DCFFET (Figure 1b,c and Figure 2). The central straight microfluidic channel surrounded by PDMS channel sidewalls is filled with saline solution. The initial values selected for various experimental parameters are as follows: σ_f_ = 10^−3^ S/m, C_s_ = 0.8 F/m^2^, λ_D_ = 37.64 nm, σ_free_ = −0.0001 C/m^2^, ε_ins_ = ε_PDMS_ = 2.8ε_0_, V_G1_ = −V_G2_ = 1500 V, Vs = 30 V, *f* = 10 kHz, L_c_ = 2.5 mm, W_c_ = 200 μm, W_ins_ = 10 μm, L_G_ = 100 μm. The thin double layer approximation used herein is reasonable by λ_D_/L_G_ = 3.76 × 10^−4^ < 0.001.

Inserting this parameter space into the scaling equations, we obtain the typical values of $\left|\zeta_{\mathrm{fixed}}\right|$ = 5.31 mV, $\left|\zeta_{\mathrm{induced}}\right|$ = 197.6 mV, $\left|u_{\mathrm{DCEO}}^{\mathrm{pump}}\right|$ = 45.17 μm/s, $u_{\mathrm{ICEO}}^{\mathrm{mixing}}$ = 1679.6 μm/s and *α* = *β* = 37.188 at the micrometer dimension. The induced zeta potential is dozens of times larger than the native zeta potential, resulting in sufficiently large turbulent ICEO vortex flow in the transverse direction compared to DCEO pump fluid motion along the channel length direction. Although the induced potential is about eight-times the thermal voltage $\left|\zeta_{\mathrm{induced}}\right|\approx 8V_{T}$ ($V_{T}\approx 25\;\mathrm{mV}$), electrochemical polarization is still in the weakly nonlinear regime of diffuse charge dynamics [61,62], so that linear asymptotic analysis under the Debye–Huckel limit is still a good approximation for the current situation.

In the device configuration of B-DCFFET, $u_{\mathrm{DCEO}}^{\mathrm{pumping}}$ is responsible for delivering fluid samples along the horizontal direction towards the downstream channel exit, and $u_{\mathrm{ICEO}}^{\mathrm{mixing}}$ assists in sample mixing in the transverse direction, both of which intersect vertically with each other. On the one hand, a lower $u_{\mathrm{ICEO}}^{\mathrm{mixing}}$ provides more time for sample nanoparticles to pass through the straight channel, that is it can generate a high throughput at the channel exit at the price of degrading the mixing efficiency. On the other hand, a higher $u_{\mathrm{ICEO}}^{\mathrm{mixing}}$ propels the fluids more intensely along the channel width direction, which tends to reinforce the mixing efficiency, while it may reduce the throughput at the channel exit by making the fluid flow pause for a longer time adjacent to the array of G terminals. 

For these reasons, the two electroosmotic flow components simultaneously produced in the flow-FET, $u_{\mathrm{ICEO}}^{\mathrm{mixing}}$ and $u_{\mathrm{DCEO}}^{\mathrm{pumping}}$, exert completely distinct effects on electroconvective pumping and mixing by B-DCFFET. What is more, a different combination of physicochemical and geometrical parameters may impose an influence on $u_{\mathrm{ICEO}}^{\mathrm{pump}}$ and $u_{\mathrm{ICEO}}^{\mathrm{mix}}$ to different extents, which is of great importance for the ultimate mixing performance of the current microfluidic device. Consequently, we then prefer to conduct direct numerical simulation to realize an in-depth comprehension of the dual functionalities of B-DCFFET with the help of the control variate method.

### 3.1. Effect of the Length of the Gate Electrode on the Device Function 

First and foremost, it is necessary for us to investigate the effect of the span of an individual G terminal on the device efficiency, in that the most salient feature of B-DCFFET introduced in the current work is embodied in the usage of face-to-face oppositely-polarized gate electrodes, rather than the single-polarity G terminal applied in conventional flow-FET.

At first, we merely switch on the DC voltage difference across the source-drain terminal and leave the two external gate terminals floating in potential. According to the scaling equations in Equations (19) and (20), the lateral vortex turbulence of ICEO vanishes, while DCEO plug flow remains unchanged and pumps the chemical analyte constantly along the channel length direction toward the outlet port. Under this situation, the simulation results indicate the surface-averaged inlet flow velocity due to the action of DCEO to be about 47 μm/s, which is in good accordance with the previous analytical approximation $\left|u_{\mathrm{DCEO}}^{\mathrm{pump}}\right|$ = 45.17 μm/s. 

As shown in Figure 2a, the mixing performance at the exit attains merely 27.8%, in the presence of the DCEO-induced pump flow rate of 47 μm/s, while in the absence of lateral ICEO turbulence. In this sense, the situation with zero gate voltages is in effect a micropump, but not a micromixer. The sole mechanism for mixing under such a condition is the molecular diffusion effect, or in other words, diffusive mass transfer across the phase interface between two co-flowing laminar streams of a transversal concentration gradient, resulting in a quite humble mixing efficiency γ = 27.8% at the outlet port. Though the V_G_-free device fails in mixing, it erects a reference state for evaluating the efficiency of active mixing with a finite value of the gate voltage.

On the contrary, turning on the face-to-face bipolar gate terminals with V_G1_ = −V_G2_ = 1500 V, induced double-layer dispersion occurring at liquid/membrane interface actuates sufficiently strong nonlinear electroosmotic slip on the channel sidewalls. ICEO streaming flow behaves as one dominating counterclockwise whirlpool across the channel width direction in the vicinity of bipolar gate electrodes, owing to the phenomenon of electric field leakage across the membranes on both sides. The field-induced ICEO vortex turbulence effectively adds to the traditional DCEO plug flow, giving rise to helical streamlines in the mixing region while straightforward streamlines in the non-mixing area, as shown in Figure 2b. Accordingly, the electrokinetics-driven flow profile from numerical simulation (Figure 2b) is in good agreement with the vivid qualitative prediction from the physical argument in Figure 1b,c.

This type of lateral turbulent ICEO vortex can bring about great benefits to engender sample mixing in microfluidics (γ = 72.81% in Figure 2b). At the same time, a net pump flow $u_{\mathrm{DCEO}}^{\mathrm{pump}}$ from DCEO delivers fluids in the direction of the background electric field toward the downstream exit. Therefore, as previously mentioned, the nondimensional number $\beta =u_{\mathrm{ICEO}}^{\mathrm{mixing}}/u_{\mathrm{DCEO}}^{\mathrm{pumping}}$ dictates the preferential functionality of B-DCFFET, which has the propensity of being a micropump or a micromixer with a smaller or larger value of *β*. 

As shown in Figure 2c, a longer gate electrode is more favorable to improve the device mixing performance. In fact, as the electrode length increases from 50 μm to 400 μm, the DCEO pump velocity remains constant at 47 μm/s (Figure 2d), since the native zeta potential is independent of the area of the polarized region. At the same time, however, the mixing fluid motion from the lateral ICEO flow field is monotonously enhanced from 430 μm/s to 560 μm/s (Figure 2d), so that the value of β rises from 9.15–11.91 with increasing coverage area of bipolar G terminals. As a consequence, the mixing index from B-DCFFET becomes higher from 64.5% to 81.9% as the gate size increases for reinforcing the internal EHD vortices (Figure 2d), while not at the price of suppressing the electrokinetic pump flow rate. 

On account of a low electrical polarizability of the PDMS sidewalls, severe leakage of electric field lines occurs throughout the dielectric membrane, resulting in an inhomogeneous distribution of relatively small induced zeta potential along the solution/membrane interface (Figure 2e), which abides by the physical perspective of ICEK presented by Bazant and Squires [30]. The induced Debye screening charge merely becomes evident next to the G terminal. That is, the polarizable surface area for actuating ICEO turbulence would be greatly enlarged as the length of G terminals gets bigger (Figure 2e), and correspondingly, the magnitude of mixing flow increases to a great extent (Figure 2d). In practical experiments, in order to relax the consumption of noble metal materials, we preferentially select an intermediate gate length of L_G_ = 150 μm in subsequent analysis. 

It is noteworthy that, whatever the gate length is, the diffusing phase interface between co-flowing electrolytes follows a zigzag course in the mixing region due to the combined action of $u_{\mathrm{DCEO}}^{\mathrm{pump}}$ and $u_{\mathrm{ICEO}}^{\mathrm{mix}}$, while it translates straightforwardly to moving into the channel exit at the downstream sections because of the sole EHD drag force from $u_{\mathrm{DCEO}}^{\mathrm{pump}}$. Accordingly, the nature of electrokinetic flow driven by B-DCFFET is to spawn simultaneous pumping and mixing without any moving parts and/or human intervention, and the dominant device functionality can be adjusted with convenience by exploiting bipolar field-effect-tunable electroosmosis control in microfluidics. 

### 3.2. Effect of Gate Voltage 

**Figure 3:**
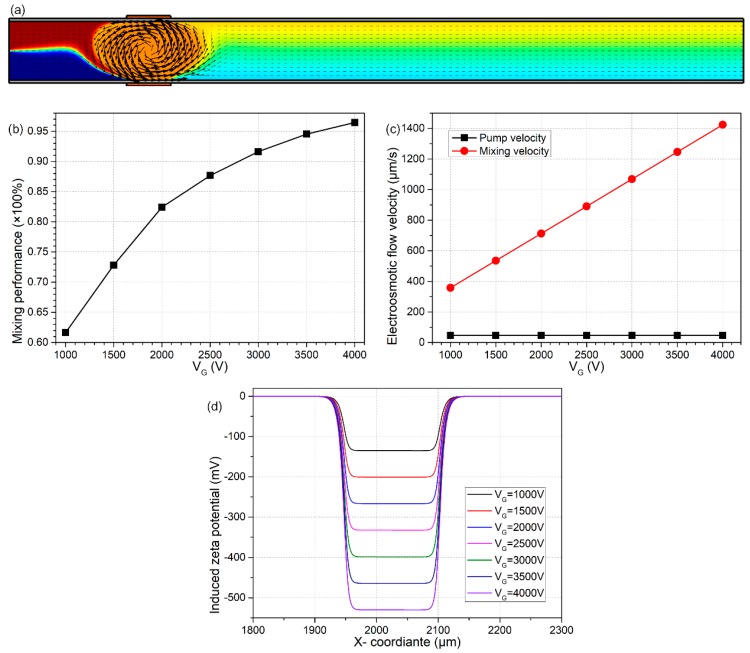
Effect of the magnitude of gate voltage V_G_ on the device performance, for given values of L_G_ = 150 μm and Vs = 30 V. (**a**) An arrow and surface plot of electroosmotic streamlines and the resulting molar concentration distribution of electroneutral chemical analyte (unit: mol/m^3^) under V_G_ = 2000 V of γ = 82.42%. (**b**) V_G_-dependent mixing performance. (**c**) Electroosmotic flow velocity in terms of pumping and mixing as a function of the magnitude of gate voltage V_G_. (**d**) Distribution of the non-uniform induced zeta potential along the channel sidewall adjacent to the negatively-polarized gate electrode for distinct V_G_, in which the negative sign implies a positive charge cloud within the Debye layer above the right-side G terminal.

We then pay attention to the influence of gate voltage magnitude V_G_ on the bifunctional microdevice with B-DCFFET, as the length of the G terminal is fixed at L_G_ = 150 μm, which is three-quarters of the width of the microchannel. As shown in Figure 3d, since the induced zeta potential increases with the applied gate voltage, the mixing velocity from ICEO has a linear growth trend as V_G_ becomes larger, while the pump flow rate due to DCEO is almost unaffected (Figure 3c), which produces a larger value of *β* and thereby permits better performance in fluid mixing at greater magnitudes of gate voltage (Figure 3b). 

Even so, we ought to take great care in observing the excessively large voltage drop across the diffuse double layer when huge bipolar gate voltages are imposed on the face-to-face oppositely-polarized G terminals. For instance, at V_G_ = 4000 V, the local maximum induced zeta potential is beyond 500 mV (twenty-times the unit thermal voltage), which may lead to an increased possibility of inducing electrochemical reaction and electrode corrosion, bringing about fatal effects to the structural configuration of B-DCFFET. Accordingly, we would rather prefer a lower magnitude of V_G_ = 2000 V out of the consideration of the operational stability of the bifunctional device, as shown in Figure 1a.

### 3.3. Effect of Source Voltage Magnitude

**Figure 4:**
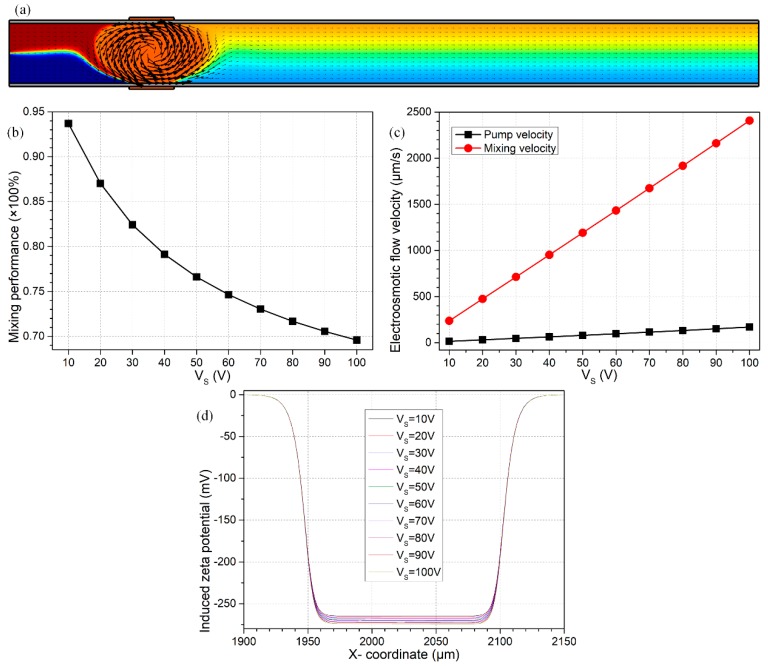
Effect of source voltage magnitude Vs on the efficiency of device bifunctionality, for given values of V_G_ = 2000 V and L_G_ = 150 μm. (**a**) An arrow and surface plot of the electroosmotic flow field and resulting analyte mass distribution (unit: mol/m^3^) under Vs = 70 V with γ = 73.03%. (**b**) Vs-dependent device mixing performance. (**c**) Characteristic pumping and mixing flow velocity of electroosmosis as a function of the source voltage Vs. (**d**) Distribution of inhomogeneous induced zeta potential at the sidewall/medium interface next to the negatively-polarized G terminal for varying Vs; the minus sign means positive charge cloud is induced inside the diffuse double layer above the right-side G terminal.

With the optimum values of L_G_ = 150 μm and V_G_ = 2000 V as found previously, we then focus on the impact of the source voltage on the device bifunctionality. Since both the native and induced Debye screening charge are forced by the background electric field E_B_ = V_S_/L_C_ into linear and nonlinear electroosmotic streaming, respectively, both DCEO pump and ICEO mixing flow components would be enhanced with an increase in the source voltage (Figure 4c). However, the longitudinal DCEO plug flow grows more quickly than the lateral ICEO turbulence (Figure 4c), in that the value of β decreases from 15.56 to 14.19 as V_S_ is elevated from 10 V to 100 V (not shown), which results in a lower mixing efficiency at a higher voltage magnitude of the S terminal (Figure 4b). Meanwhile, there is almost no variation in the induced zeta potential as Vs makes a change (Figure 4d), in good accordance with the scaling expression of Equation (17), which implies that the enhancement in transversal ICEO turbulence under this situation is not due to an improvement of the induced polarization, but is rather caused by the reinforced Coulomb force within the induced double layer in stronger tangential forcing-fields (Equation (20)).

For the above reasons, a lower magnitude of source voltage can spell much greater profit for dynamic mixing of fluid samples along the channel length direction (Figure 4b), while undesirably suppressing the forward electrokinetics-driven pump flow rate at the same time (Figure 4c). As a consequence, for the sake of reconciliation, we select an intermediate value of V_S_ to engender both a reasonable DCEO pump flow velocity u_DCEO_ = 115 μm/s and a not too bad mixing index of γ = 73.03% at the same time (Figure 4a).

### 3.4. B-DCFFET with Multiple Pairs of Face-To-Face Bipolar G Terminals

On account of producing sufficiently large DCEO streaming flow, there is a limitation in the device mixing performance under reasonable induced zeta potentials. Therefore, we next seek improved versions of geometrical configuration of B-DCFFET, in order to achieve better device bifunctionality. 

#### 3.4.1. B-DCFFET with Two Neighboring Sets of Bipolar GE Pairs

**Figure 5:**
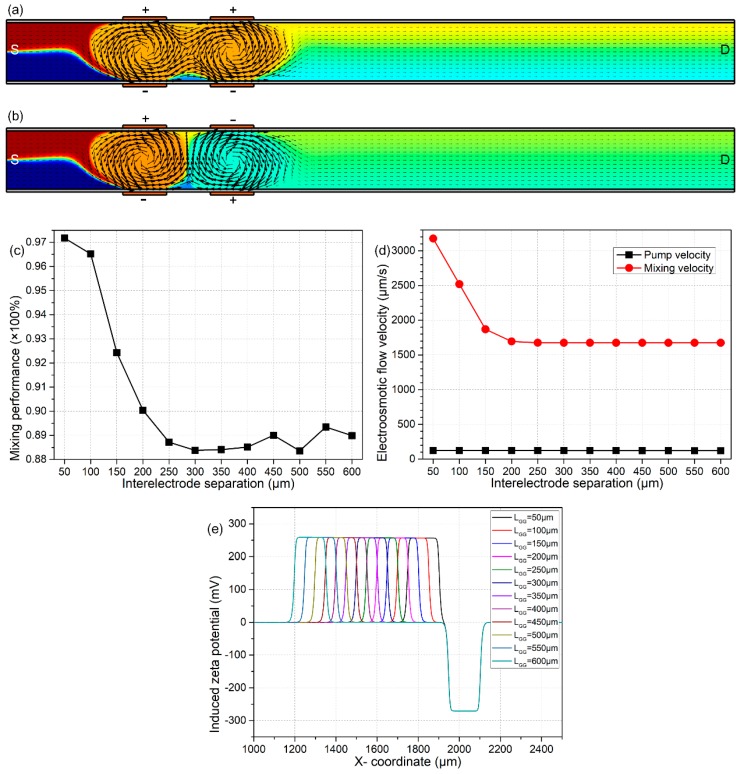
In an improved device configuration with two neighboring sets of gate electrode pairs, a numerical analysis of the effect of electrode separation L_GG_ between adjacent G terminals for given values of Vs = 70 V, V_G1_ = −V_G2_ = 2000 V and L_G_ = 150 μm. (**a**,**b**) An arrow and surface plot of DCEO fluid motion and resulting analyte concentration distribution (unit: mol/m^3^) for different combinations of external gate polarities; (a) for a neighboring GE pair of identical voltage polarity, two sequential ICEO vortexes of the same rotating directions are produced in the vicinity of the four G terminals, so that a non-ideal mixing efficiency of γ = 83.66% is obtained at the channel exit; and (**b**) for two external sets of gate electrodes of an upside-down polarity combination, an alternation in the rotating direction of the forward sample motion trajectory within the mixing region enables a much higher mixing performance of γ = 92.43% at the outlet port, which is named ‘double-inverted B-DCFFET’. (**c**–**e**) For double-inverted B-DCFFET, (c) the mixing index as a function of interelectrode gap width L_GG_, (d) L_GG_-dependent electroosmotic pump and mixing flow velocity and (e) distribution of non-uniform induced zeta potential along the planar channel sidewall adjacent to the left two gate electrodes of opposite voltage polarities for different interelectrode separations L_GG_, in which the left-side positive and right-side negative zeta potential represent the negative and positive induced counterionic charges next to the side-by-side positively- and negatively-polarized G terminals, respectively.

On the basis of flow-FET with one pair of face-to-face bipolar G terminals discussed earlier in this article, we now add another set of oppositely-polarized gate electrodes next to the first bipolar pair. As shown in Figure 5a, if the two sets of face-to-face gate electrodes are polarized in the same direction toward the right sidewall (parallel-polarization configuration), an ordinary mixing efficiency γ = 83.66% is obtained in the channel exit plane. In stark contrast, once the two sets of G terminals are reversely polarized, e.g., with the upstream set orienting toward the right sidewall, while the downstream set is pointing toward the left sidewall (anti-polarization configuration), the device performance in terms of sample mixing is greatly enhanced and attains γ = 92.43%.

The enormous disparity between the two distinct cases consists of the different electroosmotic flow field induced inside the two kinds of device configurations. The nanoparticle samples follow a completely zigzag trajectory throughout the thin liquid layer for the case of anti-polarization (Figure 5b), which can improve the device mixing efficiency to a great extent. Under the situation of parallel-polarization (Figure 5a), however, the distorted streamlines in the forward direction are merely confined to a narrow space adjacent to the two G terminals on the right side of the microchannel, which tends to inhibit sample mixing. As such, the anti-polarization B-DCFFET (Figure 5b) exhibits more excellent functionality in simultaneous electroconvective pumping and mixing than its parallel-polarization counterpart (Figure 5a). Accordingly, we should choose the priority to make use of the anti-polarization B-DCFFET for fully-automated electrokinetics-driven sample handing in microfluidics.

As shown in Figure 5c,e, the specific device performance for the ‘double-inverted B-DCFFET’ has a strong dependence on the nearest distance L_GG_ between neighboring gate electrodes on the same side. As the interelectrode separation L_GG_ increases, the electrokinetic pump flow rate keeps constant, while the lateral ICEO turbulence for sample mixing is suppressed (Figure 5d). As a result, the mixing index decays rapidly with an increase of the inter-electrode separation L_GG_ (Figure 5c). However, in real experiments, the gap size L_GG_ between neighboring G terminals cannot be made too small due to a greater difficulty in the microfabrication process, so that we select an intermediate gap distance of L_GG_ = 100 μm in subsequent analysis.

#### 3.4.2. B-DCFFET with an External Array of Face-To-Face Bipolar GE Pairs 

**Figure 6:**
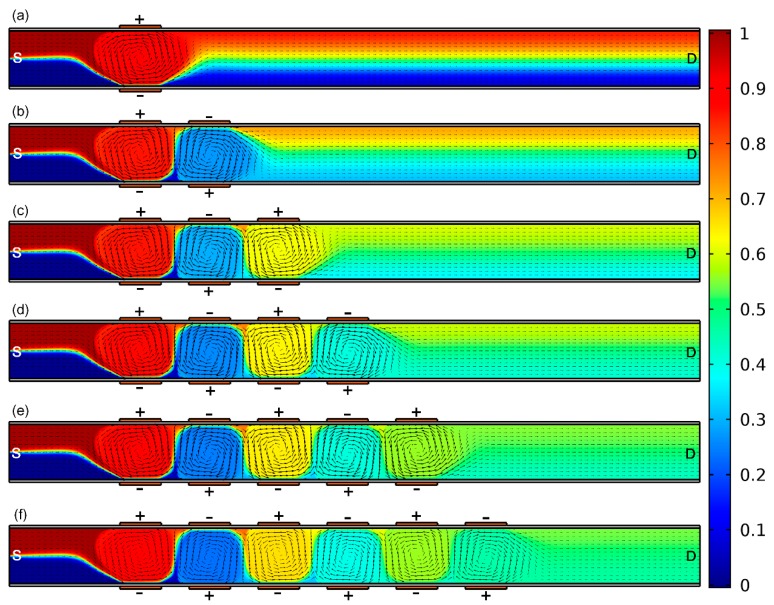
Simultaneous pumping and mixing of microflows in the device configuration with different sets of inverted B-DCFFET. (**a**–**e**) For given values of Vs = 70 V, V_G1_ = −V_G2_ = 900 V, L_G_ = 150 μm and L_GG_ = 100 μm, an arrow and surface plot of the DCEO flow field and resulting sample concentration distribution (mol/m^3^) in the integrated device design of a distinct number of G terminals; (a) single-inverted B-DCFFET of γ = 49.83%, (b) double-inverted B-DCFFET of γ = 74.38%, (c) triple-inverted B-DCFFET of γ = 85.71%, (d) quadruple-inverted B-DCFFET of γ = 88.87%, (e) quintuple-inverted B-DCFFET of γ = 92.45% and (**f**) sextuple-inverted B-DCFFET of γ = 94.79%. It is noteworthy that the highly-integrated electroosmotic pump and mixer embedded with twelve gate terminals of alternating voltage polarities exhibits optimum performance for fully-automated electrokinetics-driven analyte treatment (f), which makes it possible for us to receive almost perfect liquid mixture in the channel outlet port.

To test the feasibility of more advanced versions of B-DCFFET with multiple sets of face-to-face bipolar GE pairs of an upside-down polarity combination (Figure 6), the magnitude of gate voltage is then lowered from V_G1_ = −V_G2_ = 2000 V to V_G1_ = −V_G2_ = 900 V.

As vividly shown in Figure 6, the mixing performance from anti-polarization B-DCFFET gets better with more G terminals embedded in the straight channel on both sides. In fact, alternation times in the rotating direction of the ICEO turbulent vortex flow increase as the scope of the gate electrode array gets bigger, which well extends the length of helical streamlines along the channel length direction (or the area of mixing region) and therefore helps strengthen fluid mixing in the microdevice. In this sense, the highly-integrated ‘sextuple-inverted’ B-DCFFET embedded with twelve gate terminals of alternating voltage polarities exhibits the best performance for fully-automated electrokinetics-driven analyte treatment (Figure 6f), which makes it possible for us to receive almost perfect liquid mixture at the outlet port, even at relatively small source and gate voltages.

**Figure 7:**
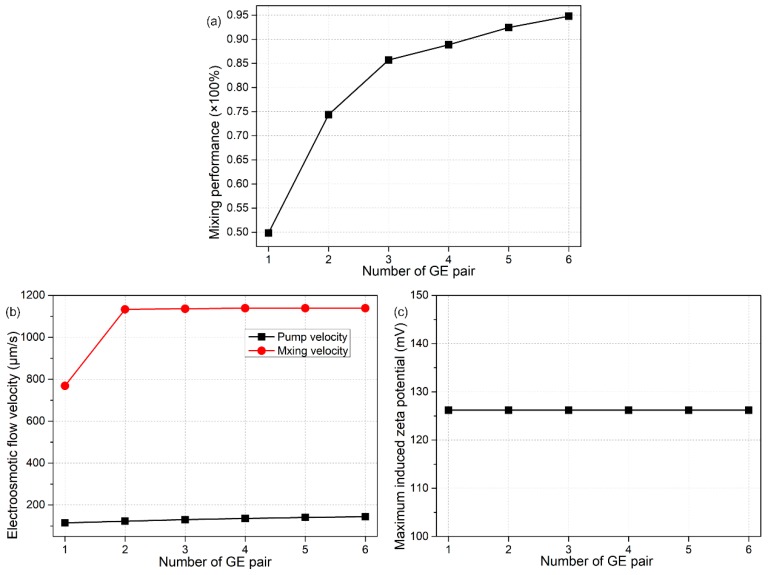
Effect of the number of gate electrode terminals (*n*) on the dual functionalities of the integrated device configuration, for given values of Vs = 70 V, V_G1_ = −V_G2_ = 900 V, L_G_ = 150 μm and L_GG_ = 100 μm. (**a**) *n*-dependent mixing performance. (**b**) Pump and mixing electroosmotic flow velocity as a function of the number of gate electrode pairs. (**c**) The largest value of induced zeta potential at the polarized liquid/membrane interface for a distinct number of G terminal pairs.

More calculation results are shown quantitatively in Figure 7, so as to illustrate in detail how the device performance makes a change as a function of the number *n* of bipolar gate-electrode pairs. As *n* increases from 1 to 6, there is more opportunity for an active interplay among the series of counter-rotating ICEO vortexes from upstream to downstream, which accelerates the lateral turbulent flow (Figure 7b) and enlarges the area of mixing region, as well (Figure 6f). At the same time, however, the DCEO pump flow rate experiences just a little bit of change at varying values of *n*. As a result, with the highly-integrated fluidic device of ‘sextuple-inverted’ B-DCFFET, we can not only get the quickest electroconvection in the forward direction (Figure 7b), but also achieve the optimal mixing efficiency as high as γ = 95% (Figure 7a). That is, the dual functionalities of B-DCFFET in simultaneous electrokinetics-driven pumping and mixing are improved at the same time with greater extension of the external G terminal array along the channel length direction. Besides, as shown in Figure 7c, the induced zeta potential is nearly independent of *n*, indicating the enhancement in mixing flow is indeed due to an effective interaction between neighboring ICEO bulk flow, while not caused by an elevated surface slip flow at the membrane/liquid interface with an increase in the number of GE pairs, which serves as the unique feature of the device configuration of a GE array. In this sense, the ‘sextuple-inverted’ B-DCFFET lays the foundation for the standard paradigm in terms of establishing bipolar flow-FET for fully electrokinetics-driven on-chip sample treatment.

### 3.5. Influence of Some Important Physicochemical Parameters

On account of its superiority, we then take advantage of the ‘sextuple-inverted’ device structure for engendering the most effective dual functionalities of B-DCFFET. Though this advanced electrode configuration (Figure 6f) has been proven by comparative analysis to be the best candidate for utilizations in a highly-integrated microenvironment, the information about the influence of several important physicochemical parameters, including the liquid conductivity, the interfacial fixed surface charge density, as well as the thickness and dielectric permittivity of the membrane, on the device performance is still missing, and therefore, it is imperative for us to conduct further simulation analyses. 

#### 3.5.1. On the Effect of Solution Conductivity 

**Figure 8:**
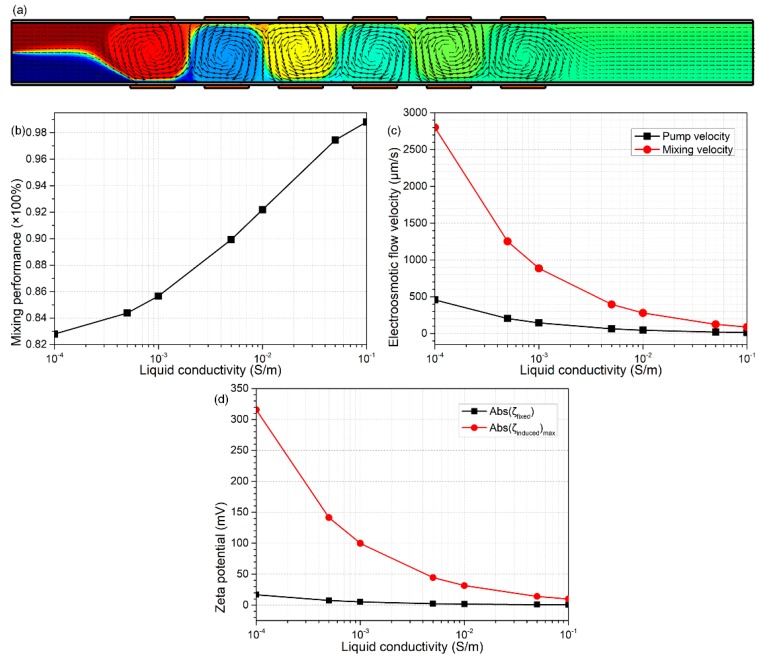
Effect of liquid conductivity σ_f_ on the device performance for sextuple-alternated B-DCFFET, for given values Vs = 70 V, V_G1_ = −V_G2_ = 700 V, L_G_ = 150 μm and L_GG_ = 100 μm. (**a**) An arrow and surface plot of the electroosmosis flow field and resulting molar concentration distribution of chemical analyte (unit: mol/m^3^) with σ_f_ = 0.005 S/m. (**b**) σ_f_-dependent mixing efficiency. (**c**) Electroosmotic flow velocity in terms of pump and mixing fluid motions at varying liquid conductivities σ_f_. (**d**) A quantitative comparison between the magnitude of native zeta potential $\left|\zeta_{\mathrm{fixed}}\right|$ and maximum induced zeta potential $\zeta_{\mathrm{induced}}^{\mathrm{maximum}}$ at the membrane/solution interface, as a function of the medium conductivity σ_f_.

An increase in medium electrical conductivity can severely shrink the Debye length of both the native and induced double layer. On the one hand, from Equation (18), with a given value of surface free charge density, the fixed zeta potential decreases with solution conductivity, due to an increase in double-layer capacitance, in good accordance with the simulation results shown in Figure 8d. On the other hand, from Equation (17), since the induced double layer is in series connection with the dielectric membrane, the impedance of the induced double layer becomes less dominating in front of that of the dielectric membrane as the ionic strength gets higher, which greatly suppresses the extension of the Debye length, resulting in a reduced induced zeta potential in concentrated buffer solutions, which agrees well with the calculation results in Figure 8d. Accordingly, both the scaling analysis and numerical simulation indicate an aggravated suppression of both electroconvective pumping and mixing flow components in the integrated device of B-DCFFET, as shown in Figure 8c. The pump flow rate almost vanishes at σ_f_ = 0.1 S/m, so the value of *β* rises to a great extent with increasing ionic strength, which causes a rather high mixing index in concentrated electrolyte (Figure 8b). However, this inflated mixing performance is at the price of sacrificing the throughput in dynamic flow conditions and therefore almost ceases to have practical value. As a consequence, the modified flow-FET with bipolar gates may malfunction in high-conductivity biological buffer solution, suggesting that we should make use of dilute electrolytes for effective electroconvective sample manipulation as much as possible in B-DCFFET. As such, we slightly raise the liquid conductivity from 0.001 S/m to 0.005 S/m for later analysis, so as to stay within the limit of dilute solution theory (Figure 8a). 

#### 3.5.2. On the Effect of Fixed Free Surface Charge Density

**Figure 9:**
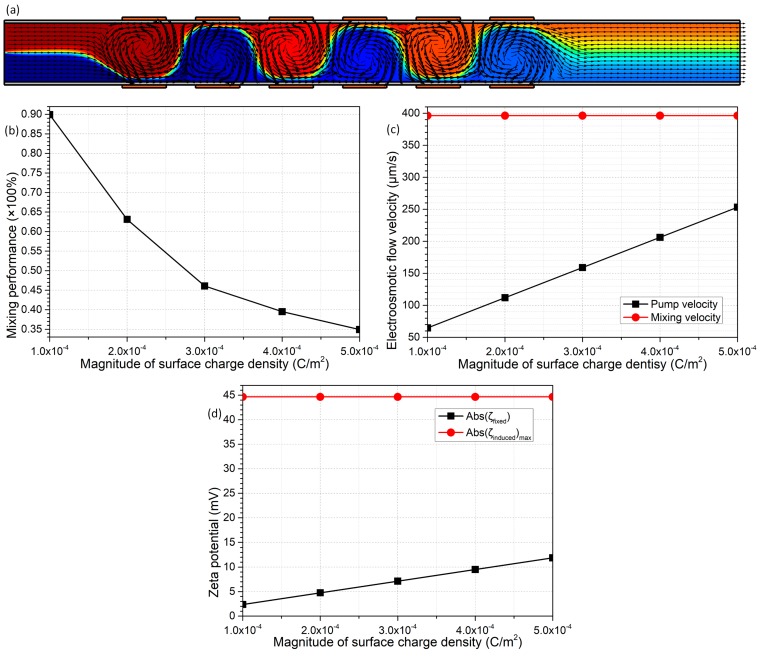
Effect of the absolute value of fixed surface charge density $\left|\sigma_{\mathrm{free}}\right|$ on the dual functionalities of the sextuple-alternated B-DCFFET device, for given values of Vs = 70 V, V_G1_ = −V_G2_ = 700 V, L_G_ = 150 μm, L_GG_ = 100 μm and σ_f_ = 0.005 S/m. (**a**) An arrow and surface plot of electrokinetic streamlines and analyte concentration distribution (unit: mol/m^3^) with $\sigma_{\mathrm{free}}$ = –0.0002 C/m^2^. (**b**) $\left|\sigma_{\mathrm{free}}\right|$ -dependent device mixing performance. (**c**) Electroosmotic pumping and mixing flow velocity for varying values of $\left|\sigma_{\mathrm{free}}\right|$. (**d**) A quantitative comparison between the magnitude of native zeta potential $\left|\zeta_{\mathrm{fixed}}\right|$ and the maximum induced zeta potential $\zeta_{\mathrm{induced}}^{\mathrm{maximum}}$ at the membrane/electrolyte interface, as a function of the magnitude of the fixed surface charge density $\left|\sigma_{\mathrm{free}}\right|$ on channel sidewalls.

By making a comparison between Equations (17) and (18), it is discovered that the fixed free surface charge density σ_free_ merely exerts an impact on the native zeta potential, while the induced zeta potential has no sensitive dependence on the magnitude of σ_free_. This simple physical justification is in perfect agreement with the simulation results in Figure 9d, where the absolute value of native potential is linearly proportional to the magnitude of σ_free_, and at the same time, the maximum induced potential keeps constant at 45 mV. Accordingly, though the DCEO pump flow rate increases from 65 μm/s to 250 μm/s with an elevation in the magnitude of σ_free_ from 10^−4^ C/m^2^ to 5 × 10^−4^ C/m^2^, the lateral ICEO turbulence for fluid mixing remains unchanged at 395 μm/s (Figure 9c). In this way, B-DCFFET of a higher free surface charge density at the liquid/membrane interface tends to be a microfluidic pump (Figure 9c) rather than a micromixer (Figure 9b). As a result, to achieve the optimal bifunctionalities for integrated B-DCFFET in simultaneous electroconvective pumping and mixing, it is our best choice to assume an intermediate value of the interfacial charge density σ_free_ = 2 × 10^−4^ C/m^2^.

#### 3.5.3. On the Effect of Membrane Properties 

**Figure 10:**
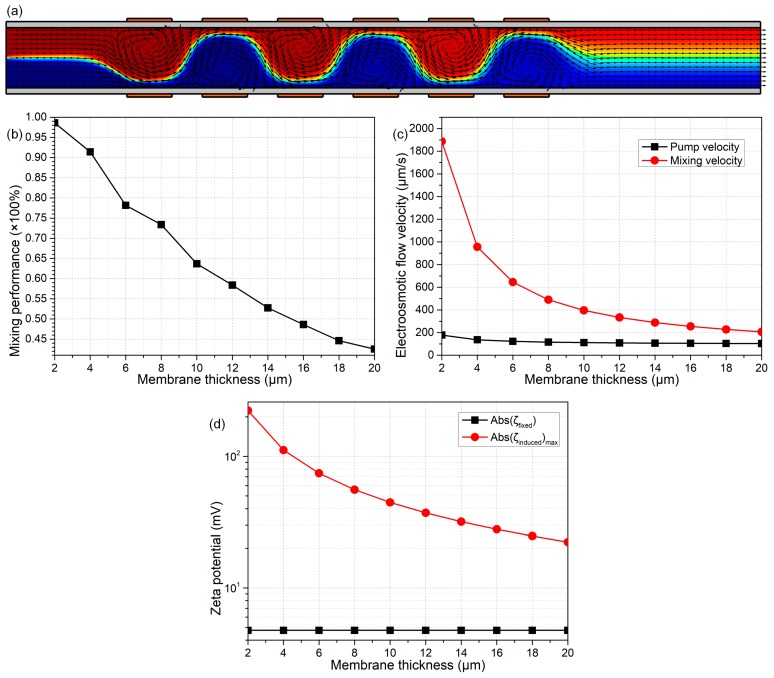
A simulation analysis of the influence of the thickness of dielectric membrane W_ins_ on the device performance of sextuple-alternated B-DCFFET, under given values of Vs = 70 V, V_G1_ = −V_G2_ = 700 V, L_G_ = 150 μm, L_GG_ = 100 μm, σ_f_ = 0.005 S/m and $\sigma_{\mathrm{free}}$ = –0.0002 C/m^2^. (**a**) An arrow and surface plot of DCEO streamlines and analyte molar concentration distribution (unit: mol/m^3^) with W_ins_ = 20 μm. (**b**) W_ins_-dependent device mixing efficiency. (**c**) Electroosmotic pumping and mixing flow velocity at different membrane thicknesses. (**d**) A quantitative comparison between the magnitude of native zeta potential $\left|\zeta_{\mathrm{fixed}}\right|$ and maximum induced zeta potential $\zeta_{\mathrm{induced}}^{\mathrm{maximum}}$ at the membrane/electrolyte interface, as a function of the membrane thickness W_ins_ of the dielectric coating layer.

**Figure 11:**
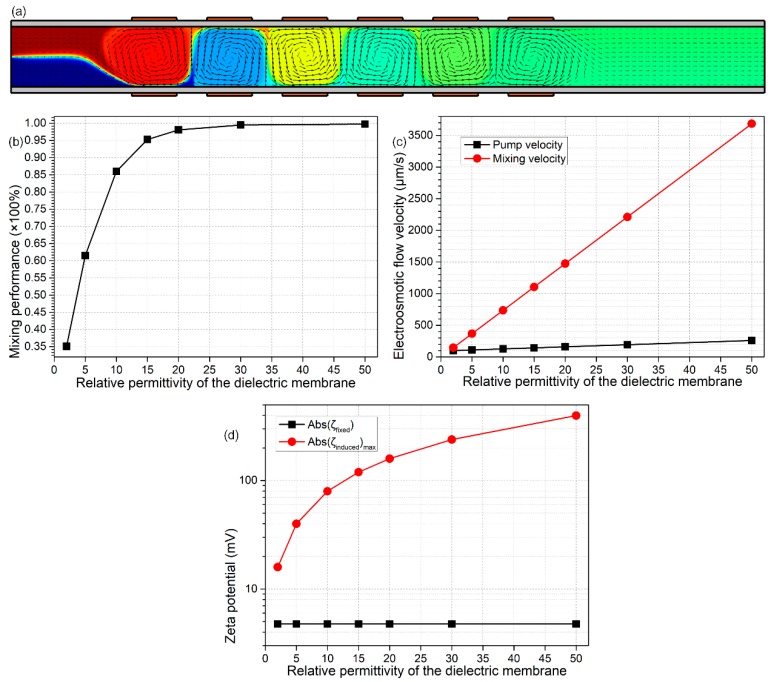
Calculation results of the influence of the dielectric permittivity of the insulation membrane ε_ins_ on the device performance of the sextuple-alternated B-DCFFET, under given values of Vs = 70 V, V_G1_ = −V_G2_ = 700 V, L_G_ = 150 μm, L_GG_ = 100 μm, W_ins_ = 20 μm, σ_f_ = 0.005 S/m and $\sigma_{\mathrm{free}}$ = –0.0002 C/m^2^. (**a**) An arrow and surface plot of electroconvective streamlines and analyte molar concentration distribution (unit: mol/m^3^) with ε_ins_ = 20ε_0_. (**b**) ε_ins_-dependent device mixing performance. (**c**) Electroosmotic pumping and mixing flow velocity for distinct membrane dielectric permittivity. (**d**) Comparison between the native zeta potential $\left|\zeta_{\mathrm{fixed}}\right|$ and maximum induced zeta potential $\zeta_{\mathrm{induced}}^{\mathrm{maximum}}$ at the sidewall/medium interface, as a function of the relative permittivity of the dielectric layer.

At the end of this work, we present the analyses about how the membrane properties affect the device dual-functionality (Figure 10a and Figure 11a). From Equation (19), DCEO streaming flow has an insensitive dependence on the membrane properties in terms of the membrane thickness W_ins_ and its dielectric permittivity ε_ins_. In stark contrast, from Equation (20), the transversal ICEO mixing fluid motion is clearly dependent on the electrical polarizability of the insulating channel sidewalls. As the membrane becomes thinner or its permittivity becomes greater, the mixing flow from nonlinear electroosmosis can be gradually enhanced (Equation (20)), and the pump fluid motion from linear electroosmosis merely has a small change (Equation (19)), in excellent accordance with the simulation results in Figure 10c and Figure 11c. The reason behind this phenomenon consists of the origin of Debye screening charge for the two distinct kinds of electroosmosis: (a) in DCEO, the counterionic charge is produced due to the fixed surface charge, while independent of the membrane polarizability and the externally-applied DC voltages (Equation (17)); (b) in ICEO, however, the interfacial charged cloud is induced by the polarization of channel sidewalls in the presence of gating field leakage. This physical justification conforms well with the theoretical prediction in Figure 10d and Figure 11d, where the induced zeta potential increases, but the native one remains unchanged with the enhanced field-induced polarizability of dielectric membranes.

In this sense, a smaller thickness and larger permittivity of channel sidewalls can improve the pumping (Figure 10c and Figure 11c) and mixing performance (Figure 10b and Figure 11b) for the device bifunctionality at the same time. Therefore, in practical experiments, it is the method of choice to take advantage of solid materials of relatively high induced polarizability to accomplish the deposition of membranes on channel sidewalls.

## 4. Conclusions

In summary, we have presented both dimensional analysis and numerical calculation, to elucidate a unique approach for actuating flexible field-effect control on electroosmosis, i.e., the bipolar DC flow field-effect-transistor (B-DCFFET). This new technique for electrokinetic manipulation of co-flowing liquid contents at the micrometer scale is originated by the Coulomb force within both the native double layer from the fixed surface charge chemical adsorbed on channel sidewalls and the field-induced counterionic Debye screening cloud due to the polarization of dielectric membranes under vertical gating fields from one pair of face-to-face oppositely-polarized G terminals embedded on both sides of the fluidic channel. Our mathematical analysis clarifies that bipolar flow-FET can engender both DCEO pumping and ICEO mixing fluid motions simultaneously in microchannels. This kind of hybrid electroconvection in DC fields is explicitly related to many exciting on-chip applications, including simultaneous electroconvective transport and stirring of fluid samples, as well as characterization of bio-particles under dynamic flow conditions in microsystems. The delicate combination of forward DCEO streamlines and transversal ICEO turbulence are even more profitable to simultaneous electrokinetics-driven delivery and mingling of sample nanoparticles, by exploiting a more advanced version of the device configuration in which an array of anti-polarization bipolar GE pairs is disposed along the channel length direction on both sides. Physical perspectives into the highly-integrated B-DCFFET presented in the current work, in the presence of a background DC field and a series of bipolar gating field leakages in perpendicular orientations, can provide utilitarian guidelines on how to establish flexible electrokinetic platforms for fully-automated analyte treatment in contemporary small-scale fluidic systems. 

However, it is still necessary for us to conduct more in-depth investigation in follow-up work. For instance, plausible stretching of current analysis in the linear regime of diffuse charge dynamics is reflected in more difficult theoretical analysis in the presence of a series of large-voltage effects, such as bipolar charge-phase-transfer reactions, nonlinear diffuse-layer capacitance, non-uniform surface conductivity within the Debye layer and the resulting concentration perturbation in the fluid bulk, as well as EHD instability from the Coulomb force within an extended space charge layer in ion-depletion zones. Under such conditions, linear asymptotic analysis breaks down, and the new mathematical model must take into account nonlinear diffuse charge dynamics when the Dukhin number is no longer negligibly small. It is our belief that bipolar field-effect flow control in static DC fields is about to benefit from considerable advances in the interdisciplinary fields of electrokinetic phenomenon and electrochemical transport in the broad context of microfluidics, nanofluidics and lab-on-a-chip technologies.

## Supplementary Materials

The following are available online at http://www.mdpi.com/2072-666X/9/2/82/s1.
