# Supplementary material for: On the Bipolar DC Flow Field-Effect-Transistor for Multifunctional Sample Handing in Microfluidics: A Theoretical Analysis under the Debye–Huckel Limit
Source: Micromachines (Basel). 2018 Feb 16;9(2):82. doi: 10.3390/mi9020082 (PMC6187470; doi:10.3390/mi9020082)
Supplement: Supplementary file 1 [file micromachines-09-00082-s001.pdf]

Supplementary Materials: On the Bipolar DC Flow Field-Effect-Transistor for Multifunctional Sample Handing in Microfluidics: A Theoretical Analysis under the Debye–Huckel Limit

Weiyu Liu, Qisheng Wu, Bobin Yao, Yanbo Li, Meng Hui, and Lin Bai

1. Analytical approximation of induced double-layer charging in the B-DCFFET device

Due to complete Debye screening on the polarizable membrane surface, the electrostatic potential right outside the Debye layer is given by:

$$\phi_f = V_s - \frac{V_s}{L_c} x \quad (S1)$$

Invoking 1D approximation, we take into account that the transversal potential gradient across the membrane thickness is much larger than its horizontal counterpart. Under this situation, the electric potential would change abruptly from the non-electrode to electrode covered area along the membrane length direction, and the boundary condition Equation (4) is reduced to the following 1D form:

$$\varepsilon_f \frac{\phi_{ins} - \phi_f}{\lambda_D} = \varepsilon_{ins} \frac{V_G - \phi_{ins}}{W_{ins}} \quad (S2)$$

From Equation (S2), the electric potential on the membrane surface can be expressed in terms of the electrostatic potential in the fluid bulk ϕ_f and the gate voltage V_G:

$$\phi_{ins} = \frac{\varepsilon_f \phi_f + \frac{\varepsilon_{ins} \lambda_D}{W_{ins}} V_G}{\varepsilon_f + \frac{\varepsilon_{ins} \lambda_D}{W_{ins}}} \quad (S3)$$

By subtracting Equation (S1) from Equation (S3), the position-dependent induced zeta potential is obtained:

$$\zeta_{induced} = \phi_{ins} - \phi_f = \left(V_S \left(\frac{x}{L_C} - 1 \right) + V_G \right) \Big/ \left(1 + \frac{\varepsilon_f W_{ins}}{\lambda_D \varepsilon_{ins}} \right), \text{ in gate-mediated area} \quad (S4)$$

$$\zeta_{induced} = 0, \text{ in non-electrode covered section} \quad (S5)$$

2. Model validation

We then conducted a series of steps of numerical calculations with different dielectric permittivity of the insulation membrane, and compared the simulation results with the analytical solution from Equation (S4) and (S5). As shown in Figure S1, the values of zeta potential plateau are on the same order of magnitude and even identical right next to the external gate terminal for the two approaches. Nevertheless, since 1D approximation is applied during the derivation of analytical

solution, there is a sudden transition of induced surface charge from zero on both sides of the gate electrode to an even summit in the intermediate region (the full line). On the contrary, since our simulation model is based on continuum mechanics, the electric field gradient varies gradually along the channel length direction, and therefore two opposite slopes appear in the vicinity of the two critical junction points, respectively (the dotted line). Even so, the analytical solution can correctly capture the global variation trend of induced counterionic charges within the Debye screening cloud for different membrane polarizability. In view of a good agreement between the two different methods of theoretical predictions, the simulation model developed in current work can work well under Debye-Huckel limit.

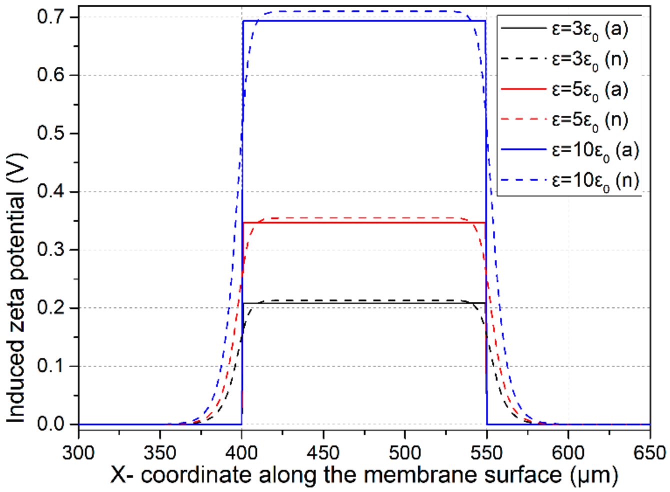

Figure S1. A comparison of distribution of induced zeta potential along the membrane surface adjacent to the external gate terminal from numerical simulation (dotted line) and analytical approximation (continuous line), with different relative permittivity of the insulation membrane, for given values of V_G = 1500 V, V_S = 30 V, W_{ins} = 10 μm.
